# Optimizing basil production and fertilizer use efficiency with consortia of plant growth-promoting bacteria

**DOI:** 10.3389/fpls.2025.1591969

**Published:** 2025-07-02

**Authors:** José I. Beltrán-Medina, Gabriela Toro-Tobón, Jonathan A. Mendoza-Labrador, Andres C. Quintero-Beyoda, Maria B. Bermudez-Cordoba, German A. Estrada-Bonilla

**Affiliations:** ^1^ Colombian Corporation for Agricultural Research (AGROSAVIA), C.I. Nataima, El Espinal, Colombia; ^2^ Colombian Corporation for Agricultural Research (AGROSAVIA), C.I. Tibaitatá, Mosquera, Colombia; ^3^ Faculty of Agricultural Engineering of the University of Tolima, Ibagué, Colombia

**Keywords:** *Ocimum basilicum* L., consortium, fertilization reduction, photosynthesis, nutrient uptake. 2

## Abstract

The basil, a widely cultivated aromatic plant, plays a crucial role in various industries but relies heavily on synthetic fertilizers, which contribute to environmental pollution. Plant growth-promoting bacteria (PGPB) offer a sustainable alternative to reduce reliance on synthetic fertilizers. In this study, three PGPB consortia and one single-strain inoculant were evaluated under reduced nitrogen and phosphorus fertilization to assess their effects on biomass production, photosynthetic efficiency, and nutritional quality. The results showed that consortium comprising *Herbaspirillum* sp., *Azospirillum brasilense*, and *Rhizobium leguminosarum*, as well as the consortium with *Rhizobium* sp. and *Azotobacter chroococcum*, significantly increased fresh biomass production–by over than 130%–compared to non-inoculated plants. Similarly, inoculation with 50% fertilization increased nitrogen and potassium uptake by over 50% compared to receiving the complete recommended fertilization without inoculation, while phosphorus uptake increased by more than 28% relative to the same control. These findings indicate that PGPB consortia offer not only an environmentally sustainable alternative to conventional fertilizers but also an effective strategy for enhancing biomass production and improving nutrient uptake in basil crops.

## 1 Introduction

Basil (*Ocimum basilicum* L. var. Nufar) is one of the most widely used aromatic plants and serves as a promising crop due to its significance in the culinary, pharmaceutical, and cosmetic industries (Carović-Stanko et al., 2010; Alhasan et al., 2020). The primary products of basil crops are dried leaves, flowers, and essential oils. Factors such as geographical origin, environmental conditions, developmental stage, soil type, and nutrition influence the chemotype of the basil plant. Selecting appropriate fertilization strategies for basil is essential for determining the quality and quantity of bioactive compounds (Makri and Kintzios, 2008; Burducea et al., 2018; Palermo et al., 2024). However, current agricultural production relies heavily on the excessive inputs of synthetic chemical nitrogen (N) and phosphorus (P) fertilizers. This overreliance contributes to soil degradation and environmental pollution, negatively impacting both environmental and economic sustainability (Nayana et al., 2024).

The availability of essential nutrients, particularly N and P, plays a critical role in plant physiology. Nitrogen is a key component of biomolecules necessary for plant development and biochemical processes. It is a determinant of light-harvesting and carbon reduction proteins, positively correlated with photosynthetic rate and performance (Mu and Chen, 2021). Conversely, P availability profoundly affects various aspects of plant photosynthetic physiology, including membrane fluidity, the generation of nicotinamide adenine dinucleotide phosphate (NADPH), and the synthesis of adenosine triphosphate (ATP) (Yan et al., 2023). Together, N and P supplies modulate light energy absorption, energy transfer, and carbon assimilation during the Calvin cycle, influencing overall plant growth and productivity (Chen et al., 2024; Ru et al., 2024).

In addition to nutrient availability, the development of plants can also be influenced by the microbial community present in the soil. Plant growth-promoting bacteria (PGPB), for instance, play a vital role in enhancing nutrient acquisition, thus optimizing the fertilizers applied. The inoculation of PGPB has become an eco-friendly strategy to mitigate the negative impact of excessive fertilization, without compromising plant production or marketability. These beneficial microorganisms colonize the root systems, fix N symbiotically and associatively (Pankievicz et al., 2019), and solubilize and mineralize nutrients such as P, potassium (K), and zinc (Alori et al., 2017). Moreover, these microorganisms can also synthesize phytohormones like auxins (3-indole acetic acid, IAA) and modulate ethylene levels (ACC deaminase), which promote root development, improve nutrient acquisition, and stimulate osmotic adjustment under stress conditions (Gupta and Pandey, 2019; Duca et al., 2014). In this respect, the combined application of PGPB and appropriate fertilization strategies can therefore maximize plant productivity while promoting sustainable agricultural practices that benefit both the environment and basil crop yield.

Our previous studies have demonstrated that single-strain inoculation and consortia of species such as *Azotobacter chroococcum, Azospirillum brasilense, Herbaspirillum* sp., *Rhizobium* sp., and *Rhizobium leguminosarum* effectively reduce N inputs in crops like cotton (Romero-Perdomo et al., 2017) and forage (Pardo-Díaz et al., 2021), and enhance P fertilization efficiency in crops such as maize (Beltran-Medina et al., 2023) and kikuyu grass (Torres-Cuesta et al., 2023). These studies highlight the potential of PGPB consortia as a promising strategy for improving agricultural efficiency, sustainability, and quality. Accordingly, this study aims to evaluate the effects of bacterial consortia and single-strain inoculation on reducing N and P fertilizer inputs in basil cultivation, as well as their impact on biomass production, plant development, physiological responses, and nutrient uptake.

## 2 Materials and methods

### 2.1 Bacterial strains and inoculants

The bacterial strains used in this study included *Rhizobium* sp. B02 (SAMN16969919), a consortium of *Azotobacter chroococcum* AC1 (B12351) and AC10 (B119352), another consortium comprising *Herbaspirillum* sp. AP21 (SAMN15498633), *Rhizobium leguminosarum* T88 (MT375980), and *Azospirillum brasilense* D7 (SAMN16830199); and a consortium of B02+AC1+AC10. All strains were provided by the Microorganism Germplasm Collection of AGROSAVIA, Colombia. For all experiments strains B02 and T88 were grown in YM culture medium (Barnet and Vincent, 1970). AC1 and AC10 were grown on MBR culture medium (Moreno et al., 2011) incubated at 30°C for 48 hours. AP21 and D7 were grown in DYGS culture medium (Baldani et al., 2014), incubated at 30°C, and agitated at 120 rpm for 48 hours. Viable cells were quantified by plate count method using the culture medium described for each strain.

Inoculation was carried out during sowing, and one week after seed emergence. The consortium were prepared by mixing equal proportions (1:1 v/v) of each bacterial strain prior to application, resulting in a final concentration of 1 × 10^8^ CFU mL^-^¹ for each strain. Inoculation was performed by drenching, with 1 mL of inoculant applied per pot. To ensure consistency across all experimental conditions, the control treatments, which did not receive inoculation, were provided with the same volume of sterile distilled water.

### 2.2 Experimental design

**Figure 1:**
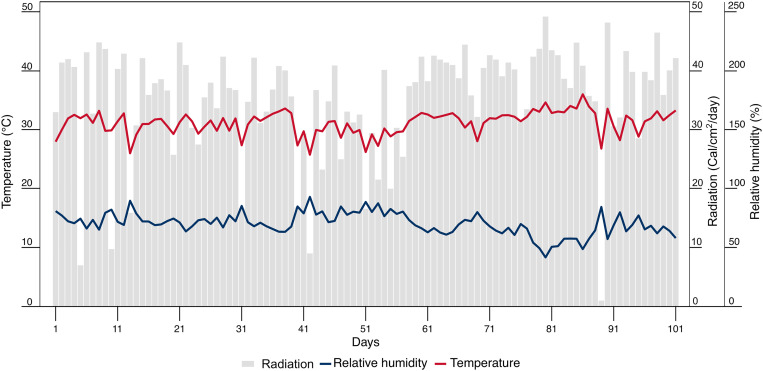
Daily climatic parameters: radiation, relative humidity, and temperature at the C.I. Nataima research center of AGROSAVIA, in Tolima, Colombia. Days were counted since the sowing of the basil seeds.

The experiment was performed at the Nataima research center of Colombian Corporation for Agricultural Research (AGROSAVIA), in Tolima, Colombia (4°11’31.65” N, 74°57’41.49” W) at an altitude of 418 meters above sea level. It was conducted under semi-controlled conditions in a greenhouse (**Figure 1**), with a mean solar radiation of 36 Cal cm^-^² day^-^¹, a mean relative humidity of 76.8%, and a mean temperature of 28.9°C.

A completely randomized 5x3 factorial experiment was conducted, comprising 15 treatments. The first experimental factor was PGPB inoculation, which included five levels: no inoculation, single-strain B02, consortium AC1+AC10, consortium B02+AC1+AC10, and consortium AP21+T88+D7. The second factor was N and P fertilization, applied at three rates: 0%, 50%, and 100% of recommended doses, using potassium nitrate and phosphoric acid as the respective N and P sources. Each treatment consisted of nine plants per experimental unit, with three different plants designated for measuring gas exchange and chlorophyll content. The experiment was independently repeated two times to ensure reliability of the results.

### 2.3 Greenhouse experiment

Basil seeds were sown into pots, containing 7 kg of nonsterile soil. The soil is classified as Inceptisol (Soil Science Division Staf, 2017) and exhibited the following physicochemical characteristics: pH (6.61), organic matter (6.20 g kg^−1^), effective coefficient for cationic exchange (3.17 cmol kg ^−1^), P (61.69 mg kg^−1^), N (1.50 g kg^−1^), K (0.31 cmol kg^−1^), S (3.58 mg kg^−1^), Ca (2.18 cmol kg^−1^), Mg (0.63 cmol kg^−1^), and Fe (21.61 mg kg^−1^). Fertilization was carried out according to the fertilization recommendation for basil cultivation, which is based on the fertigation strategy in which soluble fertilizers are supplied every three days considering the nutritional requirements of the crop and the nutrient contents at soil level. For this case, the amounts of nutrients per production cycle indicated in **Table 1** were used. Pots were irrigated at field capacity throughout the experiment.

**Table 1:** Nutrition plan used for basil cultivation by production cycle.

Macroelement	Dose (g plant^-1^)	Microelement	Dose (mg plant^-1^)
Nitrogen (N)	4.63 *	Iron (Fe)	241.76
Phosphorus (P)	0.73*	Copper (Cu)	6.93
Potassium (K)	7.61	Manganese (Mn)	13.39
Calcium (Ca)	2.13	Zinc (Zn)	15.35
Magnesium (Mg)	1.15	Boron (B)	8.47
Sulfur (S)	0.46		

*The doses of N and P were modified according to the treatments (0%, 50%, 100%).

### 2.4 Productive, physiological and nutritional parameters

The samples of productive and physiological parameters started 30 days after sowing (DAS), and every 15 days plants were sampled for 90 days, resulting in a total of 5 measurements. Each sample consisted of measuring fresh biomass, stem diameter, shoot length, SPAD units, and parameters related to photosynthetic performance. The diameter (cm) was assessed with a vernier caliper in the middle third of the stem, while shoot length (cm) was measured with a meter from the base of the shoot until the leaf primordia of the basil plant. In the middle third of a plant a mature, healthy, and fully expanded leaf was used to measure net assimilation rate (μmol CO_2_ m^-2^ s^-1^), intercellular CO_2_ concentration (μmol CO_2_ mol air^-1^), transpiration rate (mol H_2_O m^-2^ s^-1^) and stomatal conductance (mol H_2_O m^-2^ s^-1^). These measurements were assessed using the IRGA (Infra-Red Gas Analyzer) model LI-6400XT (Li-COR Inc. Lincoln, NE) from 8:00 to 11:00 h. In the same leaf we indirectly estimated chlorophyll content using a SPAD 502 plus (Konica Minolta, Inc., Osaka, Japan). At the end of the experiment, 90 days after sowing, shoot samples were oven-dried at 60°C for 72 hours for shoot tissue nutrient analysis. The N content was determined through chemical digestion using the Kjeldahl method (Nelson and Sommers, 1973), while P and K concentrations in the shoots were quantified using inductively coupled plasma–optical emission spectrometry (ICP-OES) (Wieczorek et al., 2022).

### 2.5 Statistical analysis

Data were analyzed using GraphPad Prism software (Dotmatics, San Diego, USA) and are presented as the mean ± 95% confidence interval (CI), with 9 biological replicates for physiological variables and 3 for nutrient uptake. Two-way ANOVA was performed to assess the main effects and interaction of fertilization and inoculation on fresh biomass. Normality of residuals was assessed using the Shapiro-Wilk test. Homoscedasticity was tested using Bartlett’s test. For datasets where residuals were not normally distributed or variances were heterogeneous (*P* < 0.05), the Kruskal-Wallis test was applied. When data met the assumptions of normality and homoscedasticity (*P* > 0.05), one-way ANOVA was used to assess significant differences between treatments, followed by Tukey’s *post hoc* test (α = 0.05) for multiple comparisons. For nutrient uptake, comparisons were made using Duncan’s test against the 50% fertilization without inoculation control.

To assess overall multivariate differences, Principal Component Analysis (PCA) was performed, followed by MANOVA for multivariate analysis across treatments. Outliers were identified and removed using the Robust Regression and Outlier Removal (ROUT) method, with a False Discovery Rate (FDR) set at Q = 1%. Significant differences among samples are indicated by different letters.

## 3 Results

### 3.1 Impact of N and P fertilization rates on basil development

**Figure 2:**
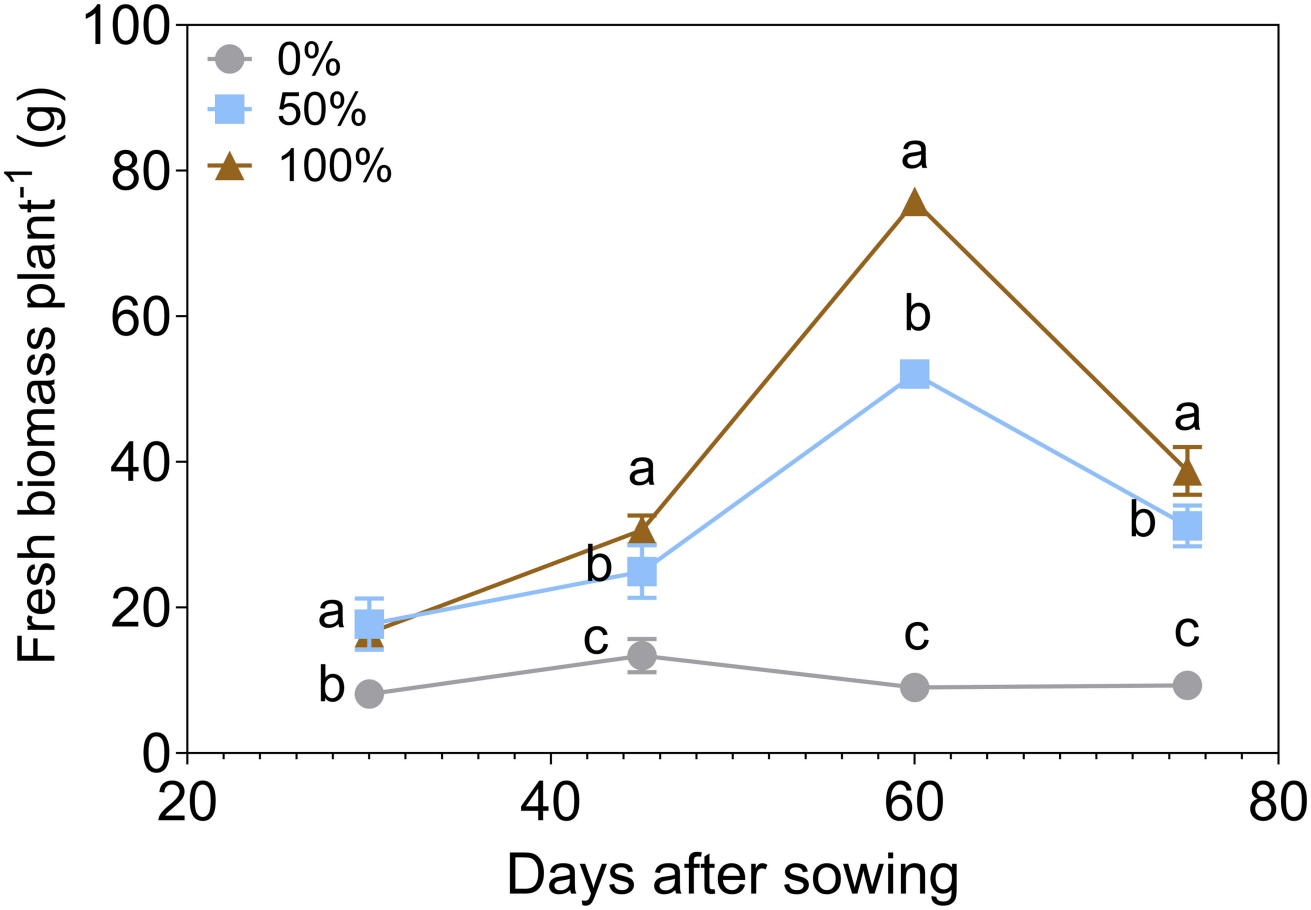
Fresh biomass per basil plant under three different nitrogen and phosphorus fertilization rates (0%, 50% 100%) (n=9). Different letters represent significant differences between treatments with Tukey’s HSD test (*P* < 0.05) for parametric data, while pairwise Wilcoxon rank-sum tests with a Bonferroni correction for non-parametric data.

The impact of N and P fertilization rates on basil was assessed at three levels (0%, 50%, and 100%) across four time points: 30, 45, 60, and 75 days after sowing (DAS) (**Figure 2**). A two-way ANOVA showed significant effects of fertilization dose (F(1.95, 77.86) = 2360, *P* < 0.0001), bacterial inoculation (F(4, 40) = 554.8, *P* < 0.0001), and their interaction (F(8, 80) = 89.29, *P* < 0.0001) on shoot dry biomass. Dose explained 63.97% of the total variation, inoculation 24.82%, and their interaction 9.68%. Biomass production increased markedly with higher fertilization rates. At 60 DAS, the 50% fertilization rate showed a fold change of 5.76 compared to the 0% rate, while the 100% rate exhibited a fold change of 8.38. At 75 DAS, the values of biomass diminished compared to 60 DAS, where the 50% fertilization treatment experienced a slight reduction over time of 40.0%, and the 100% fertilization treatment showed a more substantial reduction of 48.9%. This suggests that while initial biomass production was higher with increased fertilization, the benefits may decline over time. These findings highlight the dose-dependent effect of fertilization on basil biomass, where excessive fertilization leads to diminishing returns. This baseline provides a foundation for evaluating the impact of PGPB consortia inoculation in reducing fertilizer doses.

### 3.2 Inoculation with PGPB improves biomass production in basil

**Figure 3:**
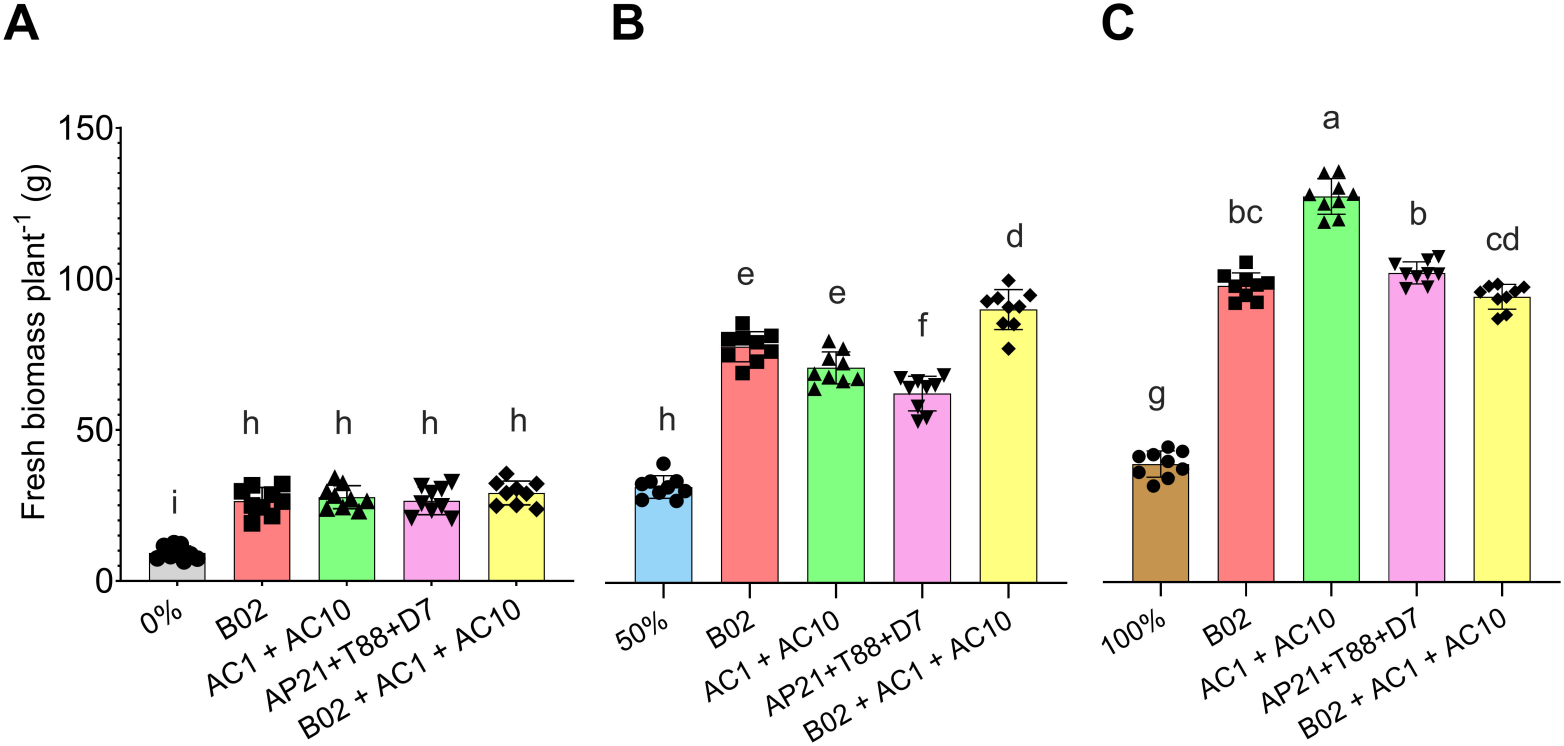
Fresh biomass per basil plant at 75 days after sowing under three nitrogen and phosphorus fertilization rates: **(A)** 0%, **(B)** 50%, and **(C)** 100%, across four microbial treatments (B02, AC1+AC10, AP21+T88+D7, and B02+AC1+AC10). Different letters indicate significant differences between treatments, based on Tukey’s HSD test (*P* < 0.05) for parametric data, and pairwise Wilcoxon rank-sum tests with Bonferroni correction for non-parametric data.

To evaluate the effect of PGPB on basil biomass production, five inoculations treatments were applied: B02, AC1 + AC10, D7 + T88 + AP21, and B02 + AC1 + AC10. After 75 DAS biomass was evaluated (**Figure 3**). PGPB inoculation significantly increased biomass production across all fertilization levels (0%, 50%, and 100%) compared to the respective non-inoculated control at each level. At 0% fertilization, inoculated plants had a 194.72% increase in biomass, while at 50% fertilization, the increase was 137.97%, and at 100%, it was 170.37%. Notably, at 0% fertilization, inoculated plants achieved biomass similar to the 50% control. Inoculated plants at 50% fertilization surpassed the biomass of the 100% control.

The highest biomass (126.90 ± 5.88 g) was observed with AC1+AC10 at 100% fertilization. However, with 50% fertilization, the B02 + AC1 + AC10 treatment (88.96 ± 6.50 g) was comparable to the 100% fertilized control (93.84 ± 4.08 g). A similar pattern was observed in shoot length and stem diameter (**Supplementary Figures S1**, **Supplementary Figure S2**). These results suggest that PGPB inoculation is more effective at lower fertilization levels, where nutrient limitations exist, and combining PGPB with 50% fertilization can achieve comparable biomass to complete fertilization.

### 3.3 Photosynthetic parameters under 50% fertilization are not affected by PGPB treatments

**Figure 4:**
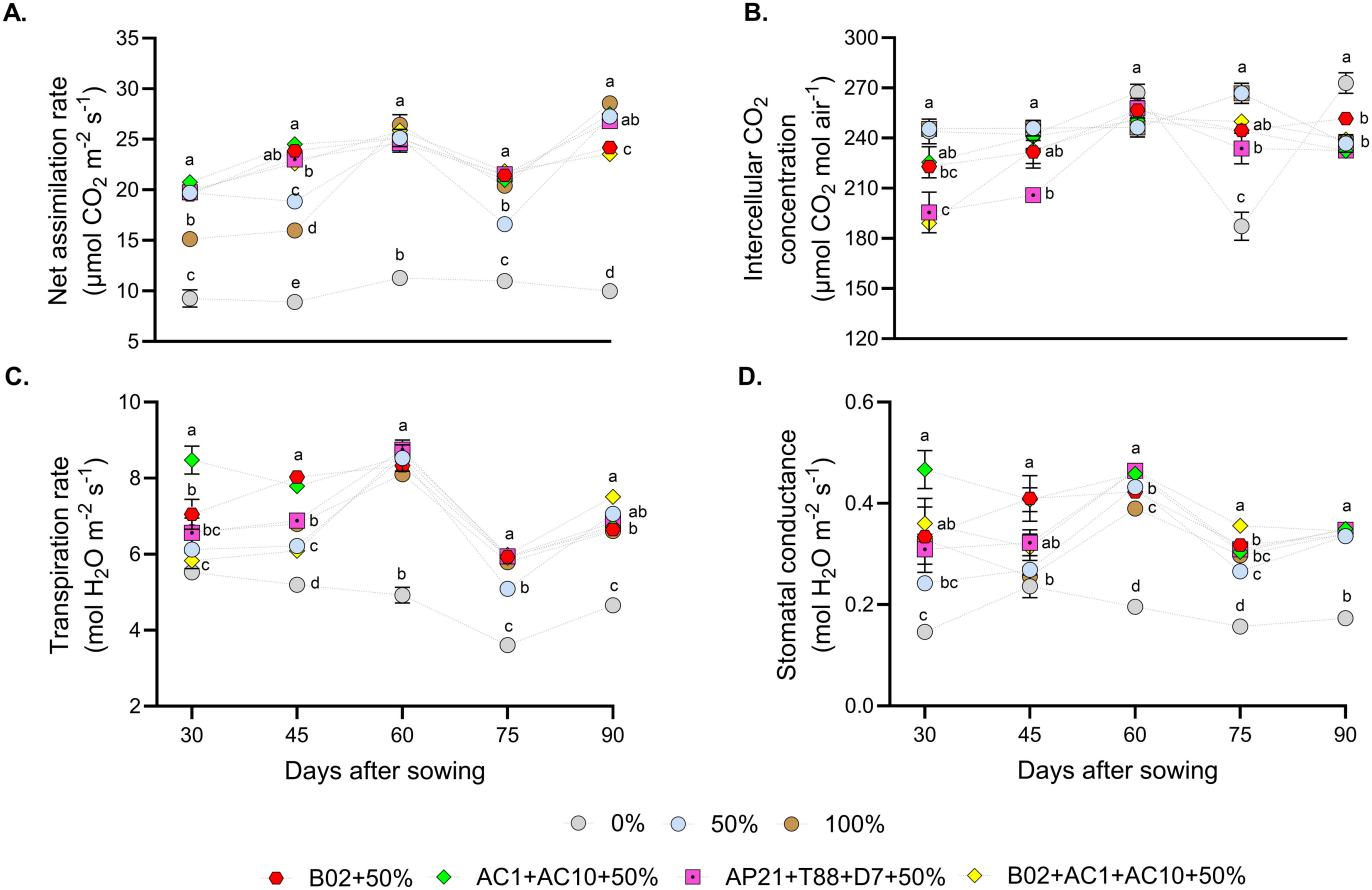
Photosynthetic parameters evaluated in basil, under three nitrogen and phosphorus fertilization rates (0%, 50%, 100%), and four different consortiums with 50% of nitrogen and phosphorus fertilization. **(A)** net assimilation rate, **(B)** intercellular CO_2_ concentration, **(C)** transpiration rate, and **(D)** stomatal conductance. Different letters represent significant differences between treatments with Tukey’s HSD test (*P* < 0.05) for parametric data, while pairwise Wilcoxon rank-sum tests with a Bonferroni correction for non-parametric data.

To evaluate the influence of PGPB and fertilization rates on basil physiological responses, photosynthetic activity was measured using an IRGA (**Figure 4**). In general, treatments with 50% fertilization combined with PGPB (single strains or consortium) showed moderate fluctuations over time. Although some statistical differences were observed among treatments at specific time points, none of the inoculated treatments or the 50% and 100% controls consistently outperformed the others throughout the evaluation period. In contrast, the 0% fertilization non-inoculated control exhibited the lowest net assimilation rate values (**Figure 4A**), and transpiration rate (**Figure 4C**), likely due to reduced stomatal conductance (**Figure 4D**). Intercellular CO_2_ levels (**Figure 4B**) remained relatively constant across treatments and time points.

**Figure 5:**
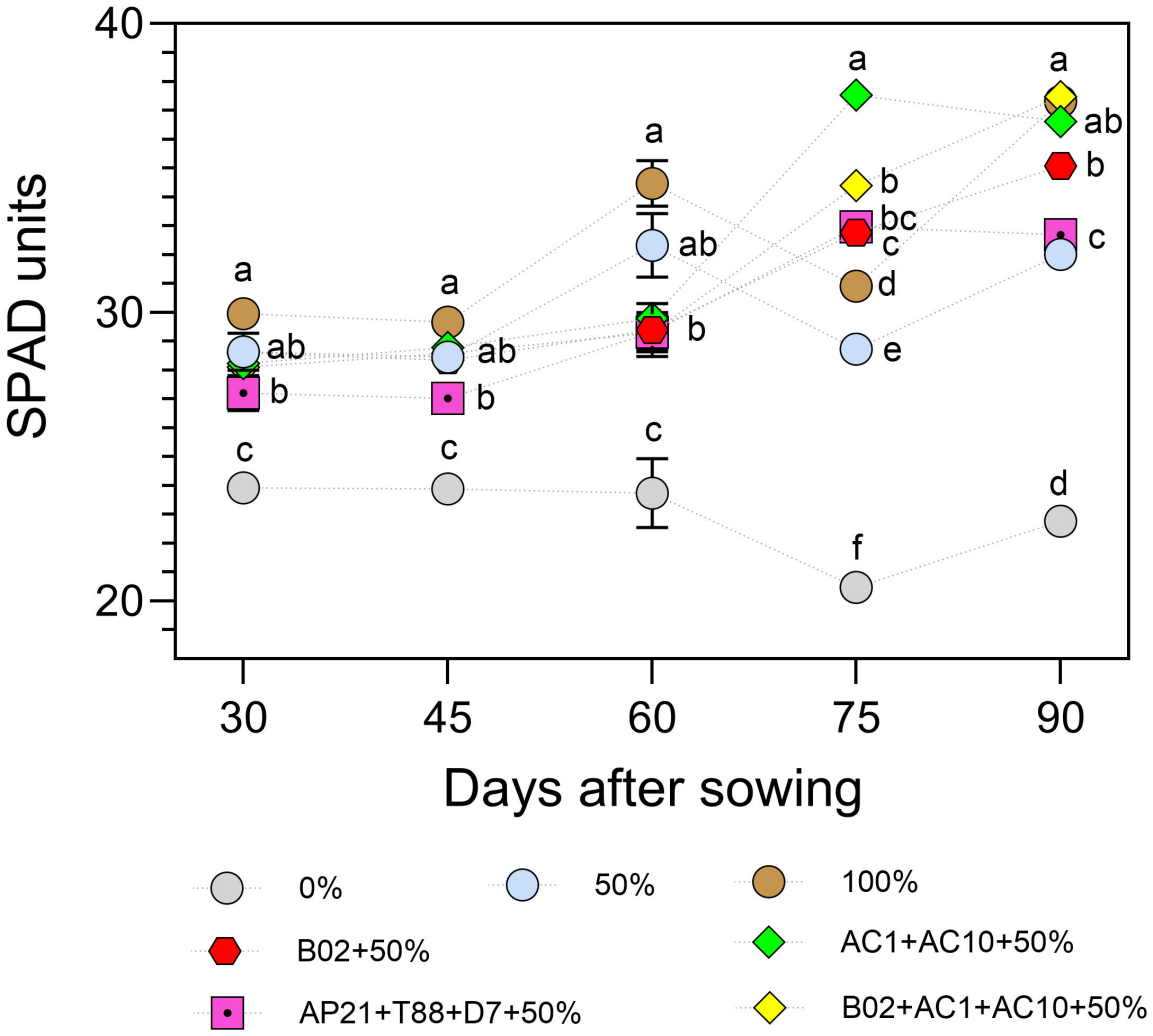
SPAD units of basil plants under three nitrogen and phosphorus fertilization rates (0%, 50%, 100%), and four different consortiums with 50% of nitrogen and phosphorus fertilization. Different letters represent significant differences between treatments with Tukey’s HSD test (*P* < 0.05) for parametric data, while pairwise Wilcoxon rank-sum tests with a Bonferroni correction for non-parametric data.

Chlorophyll content, indirectly estimated using a SPAD meter (**Figure 5**), reflects the nutritional status of the plant and influences photosynthetic activity. Between 30 and 60 days after sowing (DAS), the 100% fertilization control (non-inoculated) showed the highest SPAD values, followed by the 50% fertilization treatments, both with and without inoculation. At 75 DAS, all PGPB-inoculated treatments (single strain and consortia) showed significantly higher SPAD values compared to the non-inoculated controls at all fertilization levels (0%, 50%, and 100%). By 90 DAS, the highest pigment levels were observed in the 100% fertilization control and in the consortia B02+AC1+AC10 and AC1+AC10, followed by the single-strain B02. In contrast, the 0% control consistently exhibited the lowest values over time.

### 3.4 Inoculation with PGPB in basil plants affects crop quality

Macronutrient uptake (N, P, K) was analyzed to assess the effects of PGPB inoculation on the nutritional quality of basil under reduced fertilization conditions (**Table 2**). To evaluate the contribution of each bacterial treatment under reduced fertilization, all inoculated treatments and controls were compared to the 50% control. Inoculation with PGPB consortia significantly enhanced the uptake of key macronutrient compared to the controls (50% and 100% fertilization without inoculation). Among the treatments, plants inoculated with the consortium AP21+T88+D7 and B02+AC1+AC10 under 50% fertilization showed the highest N, P, and K uptake. Specifically, the AP21+T88+D7 treatment under 50% fertilization resulted in increases of 52.2%, 32.7%, and 63.3% in N, P, and K, respectively, compared to the 100% fertilization control. Similarly, the B02+AC1+AC10 treatment with 50% fertilization increased N, P, and K by 44.5%, 28.5%, and 55.2%, respectively, relative to the same control (**Table 2**). These results underscore the potential of PGPB inoculation to enhance nutrient uptake in basil under reduced fertilization rates.

**Table 2:** Variation of nitrogen (N), phosphorus (P), and potassium (K) uptake in basil plants growing under 50% of nitrogen and phosphorus fertilization, inoculated with three bacterial consortia and one single strain.

Treatments	Nutrient uptake		
	N (mg plant ^-1^)	P (mg plant ^-1^)	K (mg plant ^-1^)
0%	37.120 ± 1.16 e	5.653 ± 0.25 c	82.94 ± 3.21 d
50%	349.60 ± 37.05 c	6.002 ± 0.07 bc	71.64 ± 3.08 de
100%	449.00 ± 7.66 b	6.539 ± 1.05 abc	63.3 ± 6.46 e
B02 + 50%	234.10 ± 9.72 d	7.248 ± 0.56 abc	84.28 ± 4.17 c
AC1 + AC10 + 50%	450.90 ± 7.08 b	8.037 ± 1.09 ab	87.27 ± 4.46 bc
AP21 + T88 + D7 + 50%	683.30 ± 7.77 a	8.677 ± 0.41 a	103.4 ± 1.59 a
B02 + AC1 + AC10 + 50%	648.80 ± 8.73 a	8.401 ± 0.43 a	98.28 ± 0.76 ab

According to the *post hoc* Duncan test, values denoted by a different small letter differ significantly (*P* < 0.05) in a one-way ANOVA (median ± standard error).

### 3.5 Rates of fertilization and inoculation were the principal source of variability for PCA and MANOVA analysis

**Figure 6:**
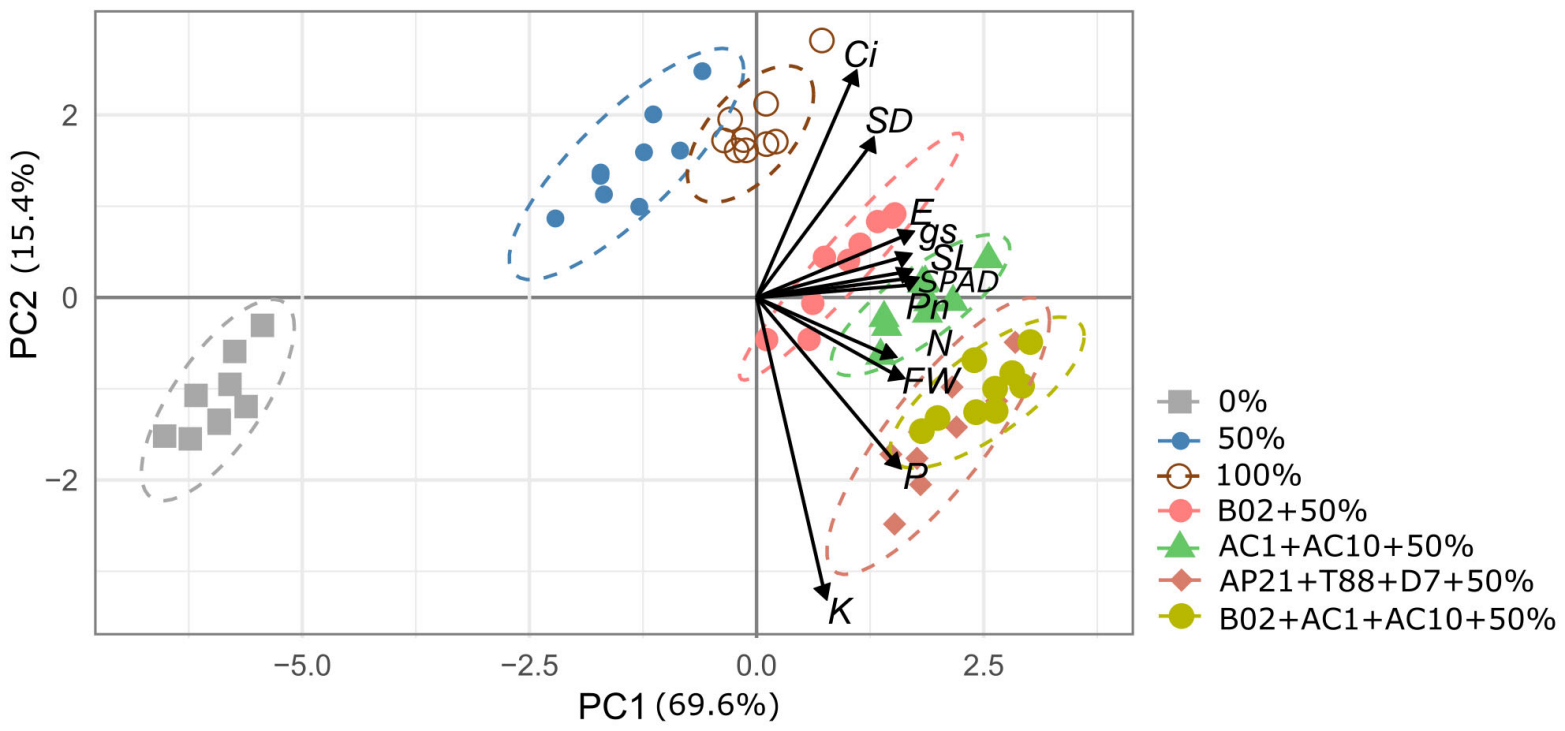
Loading and score plots from principal component analysis (PCA) of shoot length (SL), stem diameter (SD), fresh weight biomass (FW), assimilation rate (Pn), intercellular CO_2_ concentration (Ci), transpiration rate (E), stomatal conductance (g_s_), and nitrogen, phosphorous, and potassium foliar uptake (N, P, K) in basil plants subjected to 0%, 50%, and 100% N and P fertilization rates with four different PGPB inoculation.

To comprehensively understand the relationships among variables and identify patterns influenced by treatments, a principal component analysis (PCA) was performed to summarize the data and capture the key drivers of variability in the experiment. The first two principal components (PCs) (**Figure 6**) demonstrated strong correlations among the variables, with eigenvalues exceeding 1 and accounting for 85.05% of the total variance. The first component (PC1) was positively correlated with shoot length and photosynthetic parameters (Pn, E, gs, and SPAD units), while PC2 was positively correlated with intercellular CO_2_ concentration (Ci), stem diameter (SD), and P and K uptake. The 0% treatment was distinctly separated from the other two fertilization rates (50% and 100%) along PC1. The 50% fertilization treatment, inoculated with the bacterial consortium, was differentiated along PC2 and clearly clustered based on foliar nutrient uptake (P and K). Therefore, the rate of fertilization and inoculation in basil plants were key factors influencing the PCA clustering along PC1 and PC2 (**Figure 6**).

Following the PCA analysis, a MANOVA was performed to statistically validate the differences observed between treatments. The MANOVA results confirmed that the treatments had a significant effect on the measured physiological variables, with a Pillai’s trace statistic of 3.1969 and an approximate F-value of 7.6983 (*P* < 2.2e-16), further supporting the patterns seen in the PCA.

## 4 Discussion

Bioproducts based on plant growth-promoting bacteria (PGPB) offer a promising alternative to reduce synthetic fertilizer use in crop production. In this study, we tested three bacterial consortia and one single strain to enhance basil production under low N and P fertilization. Our results demonstrated that inoculation significantly increased biomass production, particularly under low fertilization conditions.

The increase in biomass can be attributed to the abilities of the strains used to solubilize and mineralize phosphates, fix atmospheric N, and produce indolic compounds and exopolysaccharides (Romero-Perdomo et al., 2017; Amaya-Gómez et al., 2020; Cortes-Patino et al., 2021; Bejarano-Herrera et al., 2024). These characteristics are commonly found in species with the potential to promote growth and mitigate damage caused by abiotic stress, such as low nutrient availability. Conversely, high N and P fertilization rates, like in the 100% fertilization, are known to suppress N fixation in the rhizosphere (Yu et al., 2019) what may alter the structure of phosphate-solubilizing microbial communities (Chen et al., 2023). Specifically, at 50% fertilization, both the single-strain B02 and the consortium AC1+AC10 produced similar fresh biomass. However, the combination of B02+AC1+AC10 outperformed the individual treatments and matched the biomass production of the B02+AC1+AC10 100% treatment. These results suggest a synergistic effect between the strains, as B02 is a phosphate-solubilizing bacteria (Beltran-Medina et al., 2023), while AC1 and AC10 are diazotrophic bacteria capable of N fixation (Romero-Perdomo et al., 2017).

Inoculation with PGPB consortia has the potential to decrease 50% of the recommended chemical fertilizer dose in basil plants. Based on this, we examined the effects of inoculation on photosynthetic performance of basil. Our results demonstrated that fertilization rate significantly influences photosynthetic processes, regardless of the inoculation consortia applied. Higher nutrient availability provides the plant with more resources for pigment production, ATP generation, stomatal regulation, and efficient translocation of photosynthesis products, thereby improving growth and biomass accumulation (Tewari et al., 2007; Mu and Chen, 2021; Liu et al., 2023). Our findings on assimilation rate (Pn), transpiration rate (E), and stomatal conductance (gs) align with reports of basil grown under optimal conditions (Barickman et al., 2021; Ciriello et al., 2024). The low photosynthetic performance observed in the 0% fertilization control, compared to 50% and 100% fertilization, was likely due to stomatal closure, consistent with other studies using varying fertilization rates (Pasquini and Santiago, 2012; Burducea et al., 2018).

75 and 90 DAS, the consortium containing AC1+AC10 strains exhibited the highest SPAD values, which reflect increased chlorophyll content. This may be attributed to their nitrogen-fixing ability (Romero-Perdomo et al., 2017) and production of indolic compounds (Lopez-Ortega et al., 2013). Indole-related molecules are known to promote plant growth by enhancing cell expansion and differentiation, regulating gene expression, and improving photosynthetic performance (Jin et al., 2023; Sun et al., 2023). Furthermore, increased N availability supports chlorophyll synthesis and other photosynthesis-related processes, thereby sustaining overall plant metabolic activity. These results are consistent with studies on basil treated with biological and conventional fertilizers (Burducea et al., 2018) and other crops subjected to different fertilization rates (Sun et al., 2020).

The use of PGPB enhanced the foliar uptake of N, P, and K in basil plants. The consortium AP21+T88+D7 and B02+AC1+AC10, combined with 50% of soluble fertilization, resulted in higher macronutrient uptake compared to the control group receiving 100% of the recommended fertilization. These results can be attributed to the ability of the bacterial species to improve nutrient availability for the plant. Introducing new bacteria into the soil can alter its physicochemical properties, affecting nutrient cycles, soil-plant interaction, and soil microbiome (Zhang et al., 2023). The bacterial consortia likely enhanced nutrient uptake by making P more available through the secretion of organic acids, phytases, or chelating agents (Cheng et al., 2023). For N, bacteria use the enzyme nitrogenase to convert atmospheric N into assimilable forms (ammonium or nitrate) (Wagner, 2011). These processes support improved nutrient uptake, which benefits plant growth and processes like photosynthesis. Our findings are consistent with previous studies that reported similar increases in nutrient content using the same bacterial species in other crops (Estrada et al., 2013; Hungria et al., 2021; Beltran-Medina et al., 2023).

Overall, our findings demonstrate that the inoculation of basil plants with *Herbaspirillum* sp. AP21, *Azospirillum brasilense* D7 and *Rhizobium leguminosarum* T88 consortium and *Rhizobium* sp. B02, and *Azotobacter chroococcum* AC1+AC10 consortium significantly enhances plant growth, biomass production, and nutritional content, even under reduced fertilization conditions. By improving nutrient uptake and supporting photosynthetic processes, these bacterial consortia offer an environmentally friendly solution to optimize crop production, contributing to more sustainable agricultural practices in basil crop. Future research should focus on exploring the long-term effects of these consortia on the microbial community and its functions, aiming to identify the specific genes involved in plant growth promotion. Additionally, it is essential to understand how plant–microbe interactions enrich the rhizosphere to enhance nutrient availability for plant uptake. These results have practical implications for farmers, offering a sustainable alternative to reduce fertilizer use while maintaining productivity in basil cultivation.

## Data Availability

The raw data supporting the conclusions of this article will be made available by the authors, without undue reservation.

## References

[B1] Alhasan A. S. Al-Ameri D. T. Al-Baldawy M. S. Abbas M. K. Hasan H. H. (2020). ‘Influence of foliar application of NPK on growth, essential oil and seed yield of Basil (Ocimum basilicum cv. Dolly). Plant Arch. 20, 2959–2962.

[B2] Alori E. T. Glick B. R. Babalola O. O. (2017). Microbial phosphorus solubilization and its potential for use in sustainable agriculture. Front. Microbiol. 8, 971. doi: 10.3389/fmicb.2017.00971 28626450 PMC5454063

[B3] Amaya-Gómez C. V. Porcel M. Mesa-Garriga L. Gómez-Álvarez M. I. (2020). A framework for the selection of plant growth-promoting rhizobacteria based on bacterial competence mechanisms. AEM. 86, e00760–e00720. doi: 10.1128/AEM.00760-20 PMC7357491 32358015

[B4] Baldani J. I. Reis V. M. Videira S. S. Boddey L. H. Baldani V. L. D. (2014). The art of isolating nitrogen-fixing bacteria from non-leguminous plants using N-free semi-solid media: a practical guide for microbiologists. Plant Soil. 384, 413–431. doi: 10.1007/s11104-014-2186-6

[B5] Barickman T. C. Olorunwa O. J. Sehgal A. Walne C. H. Reddy K. R. Gao W. (2021). Yield, physiological performance, and phytochemistry of basil (Ocimum basilicum L.) under temperature stress and elevated CO2 concentrations. Plants. 10, 1072. doi: 10.3390/plants10061072 34071830 PMC8226578

[B6] Barnet Y. M. Vincent J. M. (1970). Lysogenic conversion of Rhizobium trifolii. J. Gen. Microbiol. 61, 319–325. doi: 10.1099/00221287-61-3-319 4992272

[B7] Bejarano-Herrera W. F. Marcillo-Paguay C. A. Rojas-Tapias D. F. Estrada-Bonilla G. A. (2024). Effect of mineral fertilization and microbial inoculation on cabbage yield and nutrition: a field experiment. Agronomy. 14, 210. doi: 10.3390/agronomy14010210

[B8] Beltran-Medina I. Romero-Perdomo F. Molano-Chavez L. Gutiérrez A. Y. Silva A. M. Estrada-Bonilla G. (2023). Inoculation of phosphate-solubilizing bacteria improves soil phosphorus mobilization and maize productivity. Nutr. Cycl Agroecosyst. 126, 21–34. doi: 10.1007/s10705-023-10268-y

[B9] Burducea M. Zheljazkov V. D. Dincheva I. Lobiuc A. Teliban G. C. Stoleru V.. (2018). Fertilization modifies the essential oil and physiology of basil varieties. Ind. Crop Prod. 121, 282–293. doi: 10.1016/j.indcrop.2018.05.021

[B10] Carović-Stanko K. Orlić S. Politeo O. Strikić F. Kolak I. Milos M.. (2010). Composition and antibacterial activities of essential oils of seven Ocimum taxa. Food Chemistry. 119 (1), 196–201. doi: 10.1016/j.foodchem.2009.06.010

[B11] Chen S. Gao J. Chen H. Zhang Z. Huang J. Lv L.. (2023). The role of long-term mineral and manure fertilization on P species accumulation and phosphate-solubilizing microorganisms in paddy red soils. Soil. 9, 101–116. doi: 10.5194/soil-9-101-2023

[B12] Chen X. Zhao Y. Huang S. Peñuelas J. Sardans J. Wang L.. (2024). Genome-based identification of phosphate-solubilizing capacities of soil bacterial isolates. AMB Expr. 14, 85. doi: 10.1186/s13568-024-01745-w PMC11289785 39078439

[B13] Cheng Y. Narayanan M. Shi X. Chen X. Li Z. Ma Y. (2023). Phosphate-solubilizing bacteria: their agroecological function and optimistic application for enhancing agro-productivity. Sci. Total Environ. 901, 166468. doi: 10.1016/j.scitotenv.2023.166468 37619729

[B14] Ciriello M. Fusco G. M. Colla G. Kyriacou M. C. Sabatino L. De Pascale S.. (2024). Adaptation of basil to salt stress: Molecular mechanism and physiological regulation. Plant Stress. 11, 100431. doi: 10.1016/j.stress.2024.100431

[B15] Cortes-Patino S. Vargas C. Álvarez-Flórez F. Bonilla R. Estrada-Bonilla G. (2021). Potential of Herbaspirillum and Azospirillum consortium to promote growth of perennial ryegrass under water deficit. Microorganisms. 9, 91. doi: 10.1007/s00253-020-10869-5 33401477 PMC7824676

[B16] Duca D. Lorv J. Patten C. L. Rose D. Glick B. R. (2014). Indole-3-acetic acid in plant–microbe interactions. Antonie van Leeuwenhoek. 106, 85–125. doi: 10.1007/s10482-013-0095-y 24445491

[B17] Estrada G. A. Baldani V. L. D. de Oliveira D. M. Urquiaga S. Baldani J. I. (2013). Selection of phosphate-solubilizing diazotrophic Herbaspirillum and Burkholderia strains and their effect on rice crop yield and nutrient uptake. Plant soil. 369, 115–129. doi: 10.1007/s11104-012-1550-7

[B18] Gupta S. Pandey S. (2019). ACC deaminase producing bacteria with multifarious plant growth promoting traits alleviates salinity stress in French bean (Phaseolus vulgaris) plants. Front. Microbiol. 10.1506 doi: 10.3389/fmicb.2019.01506 31338077 PMC6629829

[B19] Hungria M. Rondina A. B. L. Nunes A. L. P. Araujo R. S. Nogueira M. A. (2021). Seed and leaf-spray inoculation of PGPR in brachiarias (Urochloa spp.) as an economic and environmental opportunity to improve plant growth, forage yield and nutrient status. Plant Soil. 463, 171–186. doi: 10.1007/s11104-021-04908-x

[B20] Jin M. Liu Y. Shi B. Yuan H. (2023). Exogenous IAA improves the seedling growth of Syringa villosa via regulating the endogenous hormones and enhancing the photosynthesis. Scientia Hortic. 308, 111585. doi: 10.1016/j.scienta.2022.111585

[B21] Liu B. Feng C. Fang X. Ma Z. Xiao C. Zhang S.. (2023). The anion channel SLAH3 interacts with potassium channels to regulate nitrogen–potassium homeostasis and the membrane potential in Arabidopsis. Plant Cell. 35(4), 1259–1280. doi: 10.1093/plcell/koad014 36653170 PMC10052404

[B22] Lopez-Ortega M. D. P. Criollo-Campos P. J. Gómez-Vargas R. M. Camelo-Rusinque M. Estrada-Bonilla G. Garrido-Rubiano M. F.. (2013). Characterization of diazotrophic phosphate solubilizing bacteria as growth promoters of maize plants. Rev. Colombiana Biotecnología 15, 115–123. doi: 10.1300/J044v13n03_10

[B23] Makri O. Kintzios S. (2008). Ocimum sp. (basil): Botany, cultivation, pharmaceutical properties, and biotechnology. J. Herbs, Spices & Med. Plants. 13 (3), 123–150. doi: 10.1300/J044v13n03_10

[B24] Moreno A. E. Rojas D. F. Bonilla R. R. (2011). Aplicación de diseños estadísticos secuenciales en la identificación de fuentes nutricionales para Azotobacter chroococcum AC1. C&TA. 12, 151–158. doi: 10.21930/rcta.vol12_num2_art:226

[B25] Mu X. Chen Y. (2021). The physiological response of photosynthesis to nitrogen deficiency. J. Physiol. Biochem. 158, 76–82. doi: 10.1016/j.plaphy.2020.11.019 33296848

[B26] Nayana H. BN M. P. Shivanna M. Halesh G. K. Prasanna H. S. MeGhana H. R. (2024). Effects of mulching and nutritional strategies on the growth and yield of sweet basil (Ocimum basilicum L.). Environ. Conserv. J. 25, 668–673. doi: 10.36953/ECJ.26422774

[B27] Nelson D. W. Sommers L. E. (1973). Determination of total nitrogen in plant material 1. Agron. J. 65, 109–112. doi: 10.2134/agronj1973.00021962006500010033x

[B28] Palermo T. B. Cappellari L. D. R. Palermo J. S. Giordano W. Banchio E. (2024). Simultaneous impact of rhizobacteria inoculation and leaf-chewing insect herbivory on essential oil production and VOC emissions in ocimum basilicum. Plants. 13, 932. doi: 10.3390/plants13070932 38611463 PMC11013597

[B29] Pankievicz V. C. Irving T. B. Maia L. G. Ané J. M. (2019). Are we there yet? The long walk towards the development of efficient symbiotic associations between nitrogen-fixing bacteria and non-leguminous crops. BMC Biol. 17, 99. doi: 10.1186/s12915-019-0710-0 31796086 PMC6889567

[B30] Pardo-Díaz S. Romero-Perdomo F. Mendoza-Labrador J. Delgadillo-Duran D. Castro-Rincon E. Silva A. M.. (2021). Endophytic PGPB improves plant growth and quality and modulates the bacterial community of an intercropping system. Sustain. Food Syst. 5. doi: 10.3389/fsufs.2021.715270

[B31] Pasquini S. C. Santiago L. S. (2012). Nutrients limit photosynthesis in seedlings of a lowland tropical forest tree species. Oecologia. 168, 311–319. doi: 10.1007/s00442-011-2099-5 21837408

[B32] Romero-Perdomo F. Abril J. Camelo M. Moreno-Galván A. Pastrana I. Rojas-Tapias D.. (2017). Azotobacter chroococcum as a potentially useful bacterial biofertilizer for cotton (Gossypium hirsutum): Effect in reducing N fertilization. RAM. 49, 377–383. doi: 10.1016/j.ram.2017.04.006 28864227

[B33] Ru C. Hu X. Wang W. Yan H. (2024). Impact of nitrogen on photosynthesis, remobilization, yield, and efficiency in winter wheat under heat and drought stress. Agric. Water Manage. 302, 109013. doi: 10.1016/j.agwat.2024.109013

[B34] Soil Science Division Staf (2017). Soil survey manual: USDA handbook No. 18 (Washington, DC: Government Printing Ofce).

[B35] Sun J. Li W. Li C. Chang W. Zhang S. Zeng Y.. (2020). Effect of different rates of nitrogen fertilization on crop yield, soil properties and leaf physiological attributes in banana under subtropical regions of China. Front. Plant Sci. 11. doi: 10.3389/fpls.2020.613760 PMC7779679 33408734

[B36] Sun P. Huang Y. Yang X. Liao A. Wu J. (2023). The role of indole derivative in the growth of plants: A review. Front. Plant Sci. 13, 1120613. doi: 10.3389/fpls.2022.1120613 36726683 PMC9885212

[B37] Tewari R. K. Kumar P. Sharma P. N. (2007). Oxidative stress and antioxidant responses in young leaves of mulberry plants grown under nitrogen, phosphorus or potassium deficiency. J. Integr. Plant Biol. 49, 313–322. doi: 10.1111/j.1672-9072.2006.00358.x

[B38] Torres-Cuesta D. Mora-Motta D. Chavarro-Bermeo J. P. Olaya-Montes A. Vargas-Garcia C. Bonilla R.. (2023). Phosphate-solubilizing bacteria with low-solubility fertilizer improve soil P availability and yield of kikuyu grass. Microorganisms. 11, 1748. doi: 10.3390/microorganisms11071748 37512920 PMC10386154

[B39] Wagner S. C. (2011). Biological nitrogen fixation. Nat. Educ. Knowledge 3, 15. Available online at: http://www.nature.com/scitable/knowledge/library/biological-nitrogen-fixation-23570419

[B40] Wieczorek D. Żyszka-Haberecht B. Kafka A. Lipok J. (2022). Determination of phosphorus compounds in plant tissues: From colourimetry to advanced instrumental analytical chemistry. Plant Methods 18, 22. doi: 10.1186/s13007-022-00854-6 35184722 PMC8859883

[B41] Yan J. Ye X. Song Y. Ren T. Wang C. Li X.. (2023). Sufficient potassium improves inorganic phosphate-limited photosynthesis in Brassica napus by enhancing metabolic phosphorus fractions and Rubisco activity. Plant J. 113(2), 416–429. doi: 10.1111/tpj.16057 36479950

[B42] Yu L. Tang Y. Wang Z. Gou Y. Wang J. (2019). Nitrogen-cycling genes and rhizosphere microbial community with reduced nitrogen application in maize/soybean strip intercropping. Nutr. Cycl Agroecosyst. 113, 35–49. doi: 10.1007/s10705-018-9960-4

[B43] Zhang Z. He H. Han T. Tian X. Pang J. Lambers H. (2023). Soil oxytetracycline alters the effects of phosphate fertilisation and Bacillus amyloliquefaciens on the bacterial community of Medicago sativa rhizosphere. Appl. Soil Ecol. 187, 104861. doi: 10.1016/j.apsoil.2023.104861

