# Supplementary material for: Optimizing basil production and fertilizer use efficiency with consortia of plant growth-promoting bacteria
Source: Front Plant Sci. 2025 Jul 2;16:1591969. doi: 10.3389/fpls.2025.1591969 (PMC12263919; doi:10.3389/fpls.2025.1591969)
Supplement: Supplementary file 1 [file DataSheet1.pdf]

Supplementary Material

Supplementary Figures:

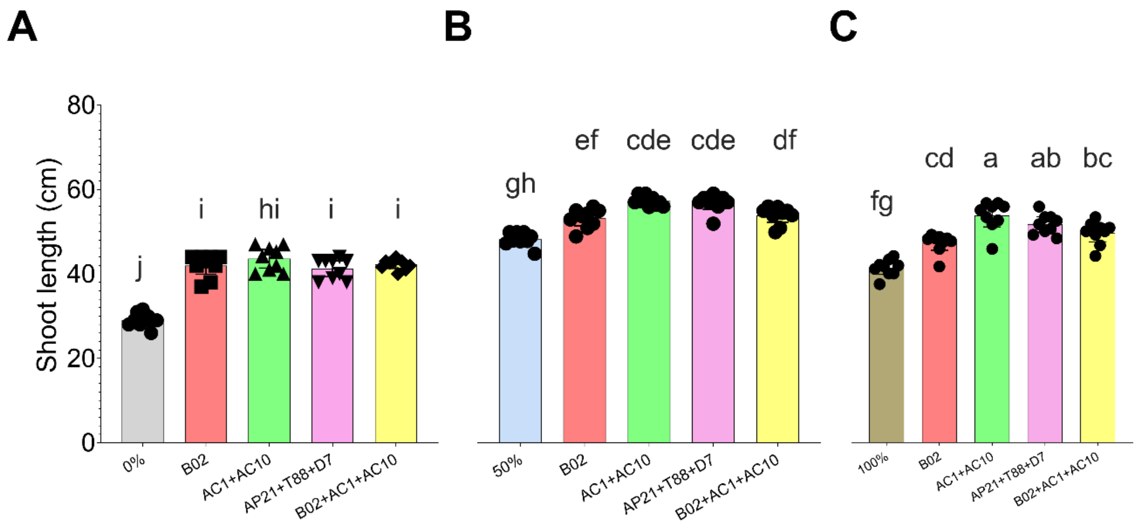

Figure S1. Shoot length of basil plants at 75 days after sowing: A) without fertilization, B) with 50% N and P fertilization, and C) with 100% N and P fertilization. And across four microbial consortia treatments (B02, AC1+AC10, AP21+T88+D7, and B02+AC1+AC10). Different letters represent significant differences between treatments with Tukey's HSD test ($P < 0.05$) for parametric data, while pairwise Wilcoxon rank-sum tests with a Bonferroni correction for non-parametric data.

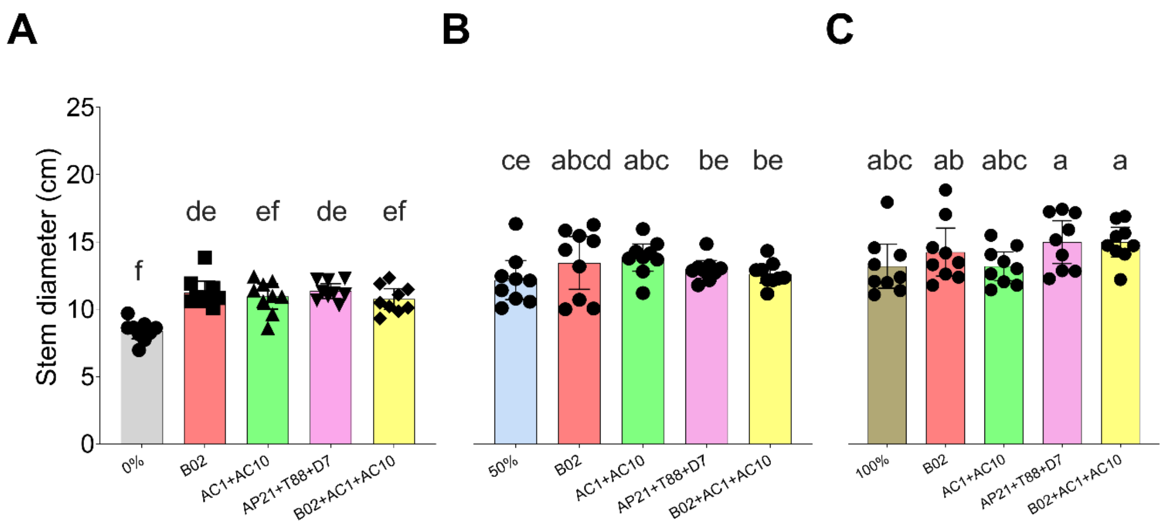

Figure S2. Stem diameter of basil plants at 75 days after sowing: A) without fertilization, B) with 50% N and P fertilization, and C) with 100% N and P fertilization. And across four microbial consortia treatments. Different letters represent significant differences between treatments with Tukey's HSD test (*P* < 0.05) for parametric data, while pairwise Wilcoxon rank-sum tests with a Bonferroni correction for non-parametric data.
